# Institutional Needs

**Published:** 1913-01-18

**Authors:** 


### Institutional Needs.

A NEW TONIC PREPARATION.

Southall Bros. and Barclay, Ltd., Birmingham, have placed on the market a new tonic preparation called "Vitafer." It is a tasteless granulated powder, the colour of cream, designed as a nutritive in cases of neurasthenia, anæmia, or in the conditions of pregnancy and lactation. The proteins of milk are its basis, in combination with certain phosphates, the tendency to constipation which often accompanies concentrated foods being corrected by the presence of magnesium. It is recommended for post-operative weakness, and may be taken with advantage in cases where an easily assimilable food is required.

